# Molecular docking analysis of HSV-1 proteins models with synthetic and plant derived compounds

**DOI:** 10.6026/97320630019981

**Published:** 2023-09-30

**Authors:** Ram Krishna, Mohammad Ajmal Ali, Joongku Lee

**Affiliations:** ICAR-Indian Institute of Vegetable Research, Varanasi-221005, Uttar Pradesh, India; Department of Botany and Microbiology, College of Science, King Saud University, Riyadh 11451, Saudi Arabia; Department of Environment and Forest Resources, Chungnam National University, Daehak-ro, Yuseong-gu, Daejeon, Republic of Korea

**Keywords:** HSV, anti-HSV drug, acyclovir, anthraquinone.

## Abstract

The atomic resolution model of US9, UL20, and gH protein of HSV is known. Hence, the ligand protein interaction of the US9, UL20, and gH protein models were carried out with synthetic drugs like acyclovir, bexarotene, vinorelbine, foscarnet, famciclovir, cidofovir and two plant derived natural drug acacetin and anthraquinone. Based on structure and docking study, it is predicted that protein US20 and gH binds with particular anti-HSV drug i.e. acyclovir, cidofovir, acacetin and famciclovir, acacetin respectively, while interaction of different protein is different with drugs.

## Background:

Herpes encephalitis, genetic herpes, herpes keratitis, and Herpes labialis, are all brought on by the herpes simplex virus (HSV). Immuno-compromised patients are more susceptible to HSV infections, which are marked by mucous membrane sores that are chronic and widespread [1]. The viral envelope of the (HSV-1, can fuse with cellular membranes to enter cells. The virus can also enter cells that are uninfected despite getting in contact with extracellular spaces by inducing virus-induced cell fusion, which enables the virus to circulate from infected to uninfected cells. It is well known that viral glycoproteins mediate these membrane fusion processes [2]. Even though viruses of the wild type only partially fuse cells, some mutations (syncytial, or syn, mutations) result in significant virus-induced cell-to-cell fusion. Most of these syncytial mutations are found in the UL20 gene [3]. It has been demonstrated that UL20 membrane protein (UL20p) is strictly necessary for virus-induced cell fusion [4]. In epithelial and neural tissues, HSV expresses the protein US9, which is crucial for virus transmission [5]. In addition, US9-HSV replicated normally in the neurons and spread there in a retrograde fashion. However, the anterograde propagation of the US9 mutant from the ganglia to the cornea was significantly restricted [6]. Consequently, US9 seems to increase viral propagation primarily in neurons. Research showing that PRV US9 increases viral glycoprotein transport in axons but not capsid transport initially came to the conclusion that US9 does not boost capsid transport [7]. Still, more recent research from the same group revealed that US9 mutants have defects in axonal transport of both capsids and glycoproteins. HSV US9 was found to be necessary for the transfer of capsids, instead of viral glycoproteins, across the retina to the optic nerve [8], according to a mouse retina model of HSV infection. The protein was initially identified in investigations on HSV US9 as a component of the viral tegument [9]. Therefore, it is of interest to document the molecular docking analysis of HSV-1 proteins models with synthetic and plant derived compounds.

## Materials and Methods:

In the present research, several three-dimensional model structures of the HSV-1's US9, UL20, and gH proteins have been developed. Ramachandran plots of PROCHECK and profiles-3D scores of the discovery studio programme version 2.0 were used to validate the models. To determine whether there is any association involving the ligand and these proteins, the computational models of all the proteins have been further studied for in silico docking studies. The several methodologies used in the present research are listed below.

## Homology modelling:

Based on the proteins homology 3-D model of US9, UL20, and gH protein of HSV-1, was generated employing discovery studio modeler 2.0 version. Sequence matching and the homology search were performed during the structure modeling. Sequences of US9, UL20, and gH protein of HSV-1, were recognized using NCBI (National Center for Biotechnology Information) database. 3-D model of US9, UL20, and gH protein of HSV-1, was validated by Ramachandran plot.

## Protein simulation:

US9, UL20, and gH Protein of HSV-1, models could be further refined by CHARMm [10] in discovery studio modeler version 2.0, it offers effective mechanics and dynamics procedures for investigating the motion and energy of molecules, from little ligands to big, multi-component biological systems. The simulation made use of the CHARMm force field (Accelrys).

## Protein-ligand interaction study:

LigandFit/ LigandScore [11], was used in this investigation.

It includes:

[1] Specify the type of binding site.

[2] Produce ligand conformations through Monte Carlo experiments.

[3] Dock every conformation using rigid body energy minimization (RBM) and a grid-based energy function to align the forms of the ligand to the binding site in 24 different orientations.

[4] Keep the highest docked structure (in various postures).

[5] Use grading function to determine the optimum binding mode for every docked structure (binding affinity prediction).

## Result and Discussion:

## Structure prediction and validation:

**Figure 1:**
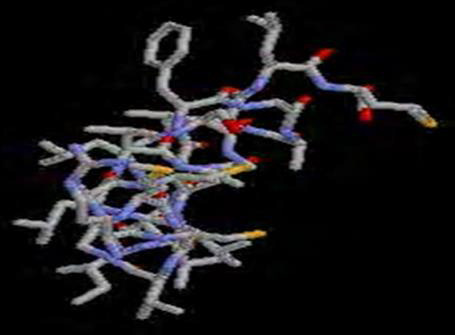
A screenshot of predicted 3D structure of US9 protein of herpes simplex virus.

**Figure 2:**
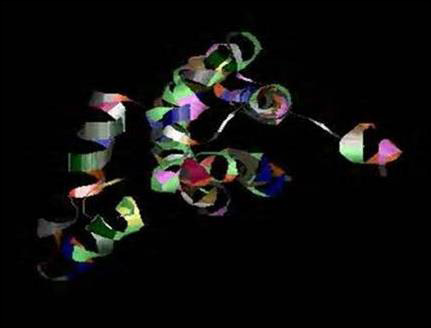
A screenshot of predicted 3D structure of UL20 protein of herpes simplex virus.

**Figure 3:**
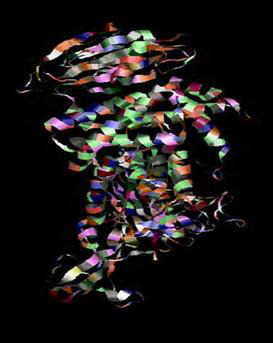
A screenshot of predicted 3D structure of gH protein of herpes simplex virus.

**Figure 4:**
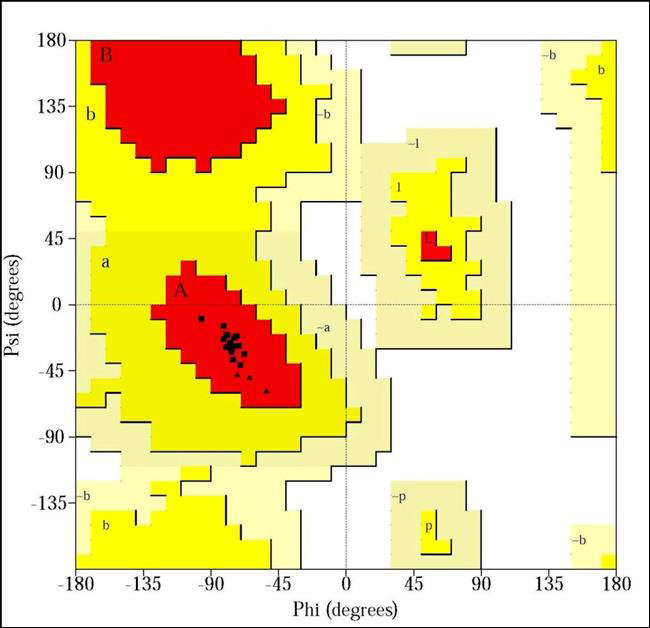
Ramachandran plot for the model of US9, protein of herpes simplex virus (100% amino acid is in most favored region).

**Figure 5:**
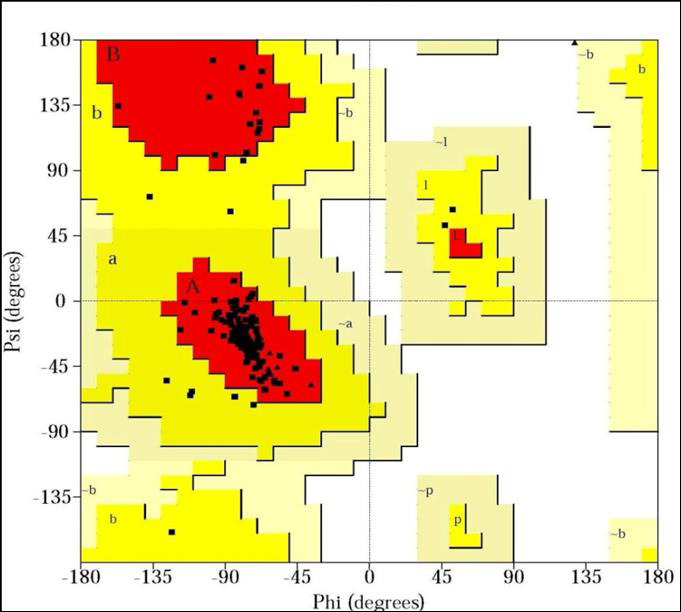
Ramachandran plot for the model of UL20 protein of herpes simplex virus (93.3% amino acid is in most favored region).

**Figure 6:**
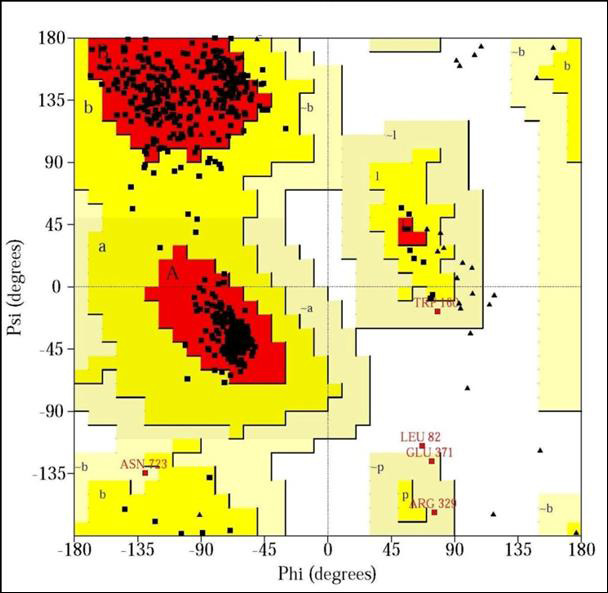
Ramachandran plot for the model of gH protein of herpes simplex virus (91.8% amino acid is in most favored region).

Distant homologues were selected for modeling US9, UL20, and gH proteins with MODELER programming. To categorize model Dali program was applied [12]. Validation of different models (Figure 1, Figure 2,Figure 3) of US9, UL20, and gH proteins was performed based on Ramachandran plot using PROCHECK which exhibited 100%, 93.3%, 91.8% residues in most favored regions respectively (Figure 4, Figure 5,Figure 6). Residues in disallowed regions was found 0.0%, 0.0% and 0.2% in the modeled structures of US9, UL20, and gH protein

## Ligand protein interaction:

Anti-HSV medications have not yet been shown to interact ligand-protein with the US9, UL20, and gH Protein of the Herpes Simplex Virus (HSV-1). LigandFit, a tool provided by DS (Accelrys), has been used to conduct a docking study for this purpose. The US9, UL20, and gH proteins of the herpes simplex virus (HSV-1) were found to have several ligand binding sites. During the investigation, various LigandFit-scored binding conformations of the ligands (anti-HSV medicines) with the protein were also found. Different ligands selected for this study includes acyclovir, bexarotene, vinorelbine, foscarnet, famciclovir and cidofovir, commonly used against HSV also two natural products showing antiherpes activity i.e. acacetin and anthraquinone. Ligandfit protocol of Accelrys Discovery Studio was employed for docking. Out of eight ligands only two were docked with protein gH and 3 with protein UL20. Acyclovir, bexarotene, vinorelbine, foscarnet, famciclovir, cidofovir, acacetin and anthraquinone, does not showed interacting potential with US9 protein of HSV-1, as it was evident from this study whivh indicates the presence of non binding site of ligands. Consequently present in silico study showed that acyclovir, bexarotene, vinorelbine, foscarnet, famciclovir, cidofovir, acacetin and anthraquinone, does not have impact on US9 protein but it has impact on RNA phase [13]. Bexarotene, vinorelbine, foscarnet, famciclovir and anthraquinone not find any ligand binding site in herpes simplex virus protein UL20, also acyclovir, bexarotene, vinorelbine, foscarnet, cidofovir, and anthraquinone didn't find ligand binding site in gH protein of herpes simplex virus. Two synthetic drugs namely acyclovir and cidofovir and one plant derived natural drug acacetin docked with protein UL20 with dock score 41.296, 5.046 and 32.589 respectively. While in case of protein gH, two drugs which include one synthetic (famciclovir) and one plant derived natural drug (acacetin) docked with dock score 4.903 and 41.721 respectively. Out of five drugs docked with protein UL20 and protein gH highest dock score found with plant derived natural product acacetin 41.721, while lowest with synthetic drug famciclovir 4.903. It is also clear both the protein docked with acacetin a plant derived natural drug with dock score 41.721 and 32.589 respectively which signifies that acacetin has high affinity for UL20 and gH protein of herpes simplex virus .

**Figure 7:**
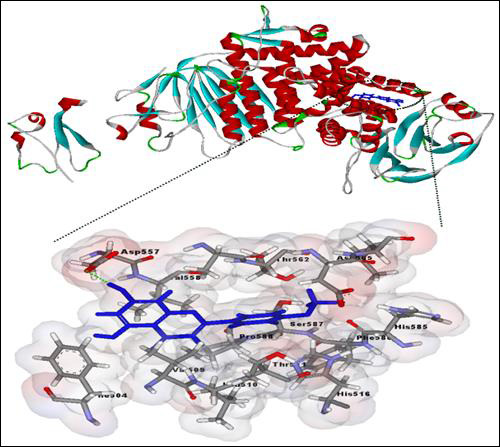
Docking screenshot of UL20 protein of *herpes simplex virus* with plant derived natural drug acacetin.

**Figure 8:**
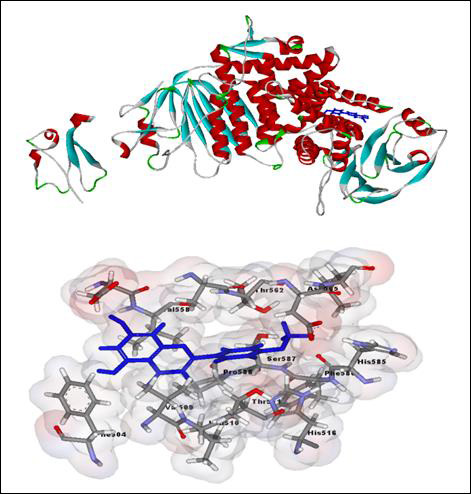
Docking screeshot of gH protein of herpes simplex virus with acacetin (with maximum docking score 41.721) with ligand receptor interaction tool of Discovery Studio (Accelrys).

Numerous research studies have reported that acacetin is a flavanoid derivative plant derived natural drug obtained from *Scoparia dulcis*, and its antiherpes activity is well reported [14]. a range of amino acids at diverse position of UL20 and gH protein were establish to be necessary for ligand protein interface with concerned anti-HSV drugs. The superimposition of acacetin with ligand binding amino acids of UL20 and gH protein of herpes simplex virus is presented (Figure 7,Figure 8). As depicted from figure Figure 7 and Figure 8 it is acknowledged that acacetin is the plant derived natural drug having affinity to UL20 and gH protein of herpes simplex virus considered in this study. Medicinal and aromatic plants play an important role in the health care of people around the world. So the advent of modern medicine derived from plants for treating human and livestock diseases is necessary need [15]. Several plants are reported to show the antiviral activities [16, 17]. Hence it is hypothesized that acacetin a flavanoid derivative of plant which is available in *Scoparia dulcis*, family, Scrophulariaceae [14], may be implicated for the treatment of HSV-1, diseases. To determine whether this novel compound is suitable for use in anti-HSV therapy, it should be studied both in vitro and in vivo.

The most significant scored (the greatest binding affinity) ligand protein interactions between the HSV-1s, UL20 and gH protein and various compounds, including anti-HSV drugs from discovery studio (Accelrys), have been taken into account (Table 1) of various docking results depending on various scoring activities. Diverse docking evaluations characterized are presented in the table are Piecewise Linear Potential-PLP1, PLP2, Jain, PMF. These evaluation methods often fall into one of two main categories, highlighting whether van der Waals, hydrophobic, or polar attractive/repulsive interactions, H-bonding interactions, or all of these interactions. The H-bonding terms in PLP (1 and 2) and the Monte Carlo scoring functions are all heavily weighted. Without H-bonding, high ligandfit scores have also been noted. Given that H-bonding seems to be crucial to the binding of various ligands. It is assumed that the ligands with dock scores greater than thirty will interact with UL20 and gH protein more favorably.

## Conclusion:

This may be the initial study of ligand-protein interaction with presently recommended drugs for disease treatments caused by herpes simplex virus and US9, UL20 and gH protein of HSV. The best inhibitory compound for UL20 and the gH protein of the HSV was determined to have the best docking score and the greatest amount of H-bonds. Acacetin has been found to be interacting with both UL20 and gH protein of different herpes simplex virus. From this UL20 and gH protein and anti-HSV drug interaction investigation, it is hypothesised that acacetin may be the best anti-HSV drug. Additionally, it has been discovered that the UL20 protein of the HSV interacts well with acyclovir. If acacetin proves to be a medication candidate for the treatment of sickness brought on by the protein UL20 and gH of the herpes simplex virus in comparison to currently used pharmaceuticals, the cost of treatment will be significantly lower.

## Funding:

Authors extend their appreciation to the Researchers Supporting Project Number (RSP2023R306), King Saud University, Riyadh, Saudi Arabia.

## Figures and Tables

**Table 1:** Different ligands protein interaction carried out in Ligand Fit tool of Discovery Studio.

**HSV-1 Proteins**	**Ligands**	**PLP1**	**PLP2**	**Jain**	**PMF**	**Dock score**
UL20	Acyclovir	76.4	67.13	1.21	60.42	41.296
	Cidofovir	57.79	64.5	1.86	37.79	5.046
	Acacetin	76.76	73.87	2.43	36.39	32.589
	Famciclovir	48.83	39.38	2.23	58.91	4.903
gH	Acacetin	48.21	50.52	2.85	38.72	41.721
